# Spontaneous Alignment of Graphene Oxide in Hydrogel during 3D Printing for Multistimuli‐Responsive Actuation

**DOI:** 10.1002/advs.201903048

**Published:** 2020-01-30

**Authors:** Mingchao Zhang, Yiliang Wang, Muqiang Jian, Chunya Wang, Xiaoping Liang, Jiali Niu, Yingying Zhang

**Affiliations:** ^1^ Key Laboratory of Organic Optoelectronics and Molecular Engineering of the Ministry of Education Department of Chemistry and Center for Nano and Micro Mechanics (CNMM) Tsinghua University Beijing 100084 P. R. China; ^2^ Beijing National Laboratory for Molecular Sciences (BNLMS) Key Laboratory of Polymer Chemistry & Physics of Ministry of Education College of Chemistry and Molecular Engineering Peking University Beijing 100871 P. R. China

**Keywords:** anisotropic composites, direct‐ink‐writing, graphene oxide, multistimuli responses, shape‐morphing

## Abstract

Natural materials are often compositionally and structurally heterogeneous for realizing particular functions. Inspired by nature, researchers have designed hybrid materials that possess properties beyond each of the components. Particularly, it remains a great challenge to realize site‐specific anisotropy, which widely exists in natural materials and is responsible for unique mechanical properties as well as physiological behaviors. Herein, the spontaneous formation of aligned graphene oxide (GO) flakes in sodium alginate (SA) matrix with locally controlled orientation via a direct‐ink‐writing printing process is reported. The GO flakes are spontaneously aligned in the SA matrix by shear force when being extruded and then arranged horizontally after drying on the substrate, forming a brick‐and‐mortar structure that could anisotropically contract or expand upon activation by heat, light, or water. By designing the printing pathways directed by finite element analysis, the orientation of GO flakes in the composite is locally controlled, which could further guide the composite to transform into versatile architectures. Particularly, the transformation is reversible when water vapor is applied as one of the stimuli. As a proof of concept, complex morphing architectures are experimentally demonstrated, which are in good consistency with the simulation results.

## Graphical abstract

A multistimuli‐responsive composite with locally controlled texture that can be printed into programmable shape‐morphing architectures is presented. The printed structures show fast, reversible, and multistimuli‐responsive shape‐morphing toward heat, light, and water (liquid and vapor).

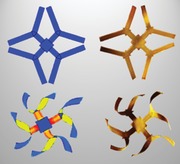

## 1 Introduction

Natural selection has endowed numerous living organisms with remarkable heterogeneity at multiple length scales by altering their local chemical compositions or exquisitely manipulating orientation of their building blocks.^1^, ^2^, ^3^ Among these organisms, anisotropic components are the key to realizing unique properties, which satisfy their specific demand of normal physiological activities in surrounding environment.^4^, ^5^, ^6^ For examples, the alignment of stiff fibrils in soft matrices, such as animal bones^2^ and animal silks,^3^ brings about extraordinary mechanical properties against structural damage, delamination, wear, and long‐term fatigue. The spatial arrangement of cellulose in many plants, such as conifer pinecone,^4^ wheat awn,^5^ and orchid tree seedpod,^6^ leads to their morphology change upon stimulation of the environment. These ingenious biological strategies provide infinite inspiration for developing heterogeneous synthetic materials with unique structures and functions, which find various applications in fields such as biomedicine,^7^ biomimetic systems,^8^ sensors,^9^ soft robotics,^10^ and flexible electronics.^11^

To create diverse anisotropic structures, versatile fillers at multilength scales, such as carbon fibers/nanotubes,^12^ nanoclay,^13^ and alumina platelet,^14^ have been introduced in homogeneous matrix. The additives enable the resultant composites with properties beyond each of the components. However, these chemically inert fillers often result in poor interfacial interactions with the surrounding matrix and inferior load transfer, thus incurring the risk of structural failure.^15^ Alternatively, graphene oxide (GO) is shown to be a superior additive because of abundant oxygen‐containing functional groups on the monolayer carbon basal surface, providing good interfacial compatibility with surrounding matrix via hydrogen bond interaction.^16^ Besides, its high aspect ratio, remarkable mechanical properties, high flexibility/ductility, and good optical/thermal properties, enrich it with great structural and functional diversity.^17^

The unique properties of natural materials usually stem from their rich structural diversity.^1^, ^2^, ^3^, ^4^, ^5^, ^6^ For example, the variant arrangement of cellulose fibrils in the thickness direction of the wheat awn causes its motion along with humidity change, which propels the seeds dispersal.^5^ In contrast, there is usually short of locally structural variation in artificial materials, which severely limits their potential for functional diversity.^18^, ^19^, ^20^ It is important to develop techniques for creating complex structures with locally controlled textures. As a state‐of‐the‐art additive manufacturing technique, direct‐ink‐writing 3D printing is promising for its bottom‐up construction of diverse custom‐designed structures.^21^, ^22^, ^23^ More importantly, the site‐specific anisotropy can be easily controlled by designing printing procedure from one region to another or applying external fields (force, electric, and magnetic field),^21^, ^22^, ^23^ which is beyond the capability of conventional 2D fabrication techniques.

Herein, we report the direct printing of spontaneously aligned GO flakes in sodium alginate (SA) matrix with site‐specific orientation, which can be fast actuated into versatile morphology upon multi‐stimulation. Owing to their good interfacial compatibility, GO flakes are uniformly distributed in the SA matrix. The GO flakes are spontaneously aligned in the SA matrix due to the large shear force when being extruded from the nozzle and then realigned horizontally after drying on the substrate as a synergetic effect of the confinement of substrate and the gravity effect, leading to the formation of a brick‐and‐mortar structure. The GO confinement restricts the deformation freedom of the SA matrix and endows the composite with spatially anisotropic deformation upon activation. By designing the printing pathway, the orientation of GO flakes in the composite can be locally controlled. When printed in a vertically stacked bilayer configuration, two kinds of shape‐morphing architectures, namely, bending and twisting, could be obtained. Directed by finite element simulation, the morphing architectures can be rationally designed and tuned. For demonstration, we constructed a series of complex architectures transformed from printed planar structures. Remarkably, the printed composite showed a fast response to multiple types of stimuli, including water, heat, and light. Particularly, reversible shape‐morphing can be realized when using water vapor as the stimulus. The fast and reversible shape‐morphing composite in response to multistimuli may shed new light in developing smart responsive systems.

## 2 Results and Discussion

### 2.1 Spatial Confinement of Aligned GO in the Hydrogel Matrix

**Figure 1:**
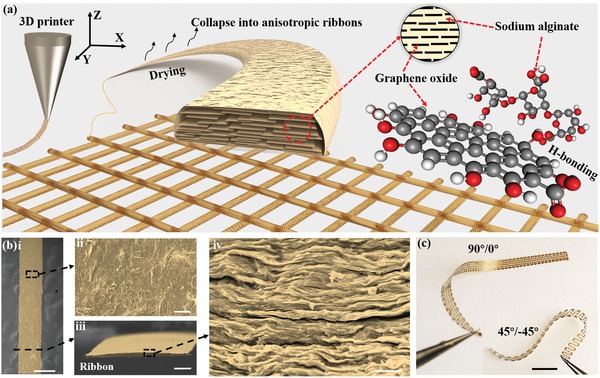
Schematic illustration of direct ink writing of GO/SA composite, structures of the printed GO/SA ribbons, and the printed strips. a) Composition of the composite and the assembling structure of the GO in the printed ribbons. b) Scanning electron microscopy (SEM) images of a printed ribbon (i, ii). SEM images of the cross section of a printed ribbon (iii, iv). The scale bars for (i)–(iv) are 500, 20, 100, and 5 µm, respectively. c) Typical examples of printed strips with 90°/0° and 45°/−45° configurations. The scale bar is 1 cm.

The printing ink includes two main components, namely, the GO filler and the SA matrix. The components and their mixtures are highly hydrophilic (see the water contact angle in Figure S1, Supporting Information), due to the existence of masses of oxygen‐containing functional groups (—O—, —OH, and —COO^−^).^24^ The addition of GO flakes brings about structural anisotropy and functional diversity into homogenous SA matrix. As illustrated in **Figure** **1**a, uniformly dispersed GO/SA composite ink is extruded from a tapered nozzle, forming an anisotropic filament and collapsing into a ribbon after drying on a substrate. Owing to the large shear force induced by the nozzle, the GO flakes in the soft SA matrix are simultaneously aligned along the axis of the filaments during extruding. After the ink is printed on substrate, the aligned GO flakes in the dilute SA matrix tend to lie flat on the substrate as a result of the confinement of substrate and the gravity effect. The extruded filaments eventually collapse into anisotropic ribbons with nacre‐like microstructures, which compose the printed planar structures (see more detailed analysis in Figures S2–S4, Supporting Information). Notably, our 3D printing method endows the printed composites with remarkably anisotropic properties along their 3D directions. Unlike previously reported nacre‐like composites with isotropic properties in their horizontal plane,^25^ the printed ribbons are anisotropy along the longitudinal and transverse direction, which can be ascribed to the selective alignment of GO flakes induced by shear force during printing (see detailed analysis in Figure S3, Supporting Information).

A printed ribbon is shown in Figure 1b(i) with some wrinkles on its surface (Figure 1b(ii)). The cross section of the ribbon shows a distinct nacre‐like structure (Figure 1b(iii),(iv)). The high alignment can be further confirmed by 2D wide angle X‐ray diffraction (2D‐WAXD) pattern with a high Herman's orientation factor of 0.78 (Figure S4c, Supporting Information). Versatile structures can be constructed by the 3D printer. Two typical bilayer strips with lattice configurations of 90°/0° and 45°/−45° are shown in Figure 1c. Solid (fully infilled) and lattice trips with different filling densities and dimensions can be easily obtained, as shown in Figure S5a,b (Supporting Information), respectively. Unless otherwise stated, the strips are all fully printed. Furthermore, the mechanical properties of the printed strips along the printing direction were investigated (Figure S6a, Supporting Information). The Young's modulus of the printed strips can be as high as 2.57 ± 0.22 GPa and gradually increases to 7.38 ± 0.31 GPa with increasing GO content (Figure S6b, Supporting Information). Notably, the strip with the printing pathway along the longitudinal direction (90°) exhibits a larger Young's modulus than that printed in the other way (Figure S6c, Supporting Information), which can be ascribed to the anisotropic arrangement of GO flakes in the SA matrix (see detailed analysis in Figures S2–S4, Supporting Information). In addition, difference can also be found in the printed strips with lattice configurations of 90°/0° and 45°/−45° (Figure S6d, Supporting Information).

### 2.2 Interfacial Compatibility of GO and SA

**Figure 2:**
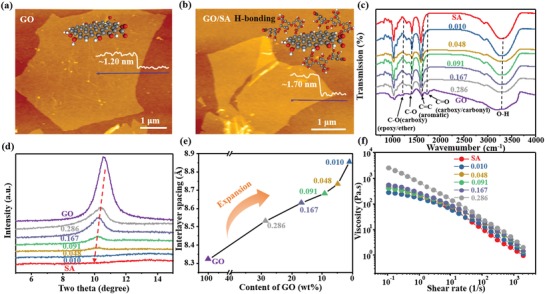
Composition and properties of the GO/SA composite ink. a) Atomic force microscopy (AFM) image of single‐layer GO flake. b) AFM images of GO/SA hybrid flake. c) Fourier transform infrared spectra of GO, SA, and their composites with various solid content of GO. d) X‐ray diffraction spectra of the printed composites with various GO contents. e) The interlayer spacing as a function of GO contents. f) Apparent viscosity as a function of shear rates for inks with various GO contents.

For a controllable anisotropy of printed filaments, GO/SA composite ink should be rationally designed. As shown by the atomic force microscopy (AFM) image in **Figure** **2**a, the GO flake has a thickness of about 1.2 nm. After mixing with SA, SA biopolymer will be absorbed on the GO flakes, which can be proved by the topography of GO/SA (Figure 2b). The average thickness of the absorbed SA is calculated to be about 0.25 nm, which is in accordance with the results of a previous report.^25^ The interaction between the GO flakes and SA biopolymer was studied using Fourier transform infrared (FTIR) spectroscopy (Figure 2c). The bands at 1728 and 1296 cm^−1^ represent the stretch of C=O and C—O, respectively, and the intensities increase along with the increase of the GO. With the increase of GO in the ink, the peak at 1296 cm^−1^ shifts to a low wavenumber at 1221 cm^−1^. Moreover, the peaks at 1406 and 1592 cm^−1^ (i.e., C—O and C=O, respectively) shift to high wavenumbers at 1408 and 1620 cm^−1^. These results indicate the formation of H‐bonds among the oxygen‐containing functional groups, which enables an excellent interfacial compatibility.^25^ Besides, X‐ray diffraction (XRD) was used to characterize the interspace between neighboring GO flakes in the printed structure (Figure 2d). The absorption of SA on the GO can induce expansion of the space between neighboring GO flakes, leading to the decreased intensity and the shift of the peak located at about 10.5°. The corresponding *d*‐spacing of the pure GO flakes is calculated as 0.83 nm, whereas that with a GO solid content of 0.01 increased to 0.89 nm (Figure 2e).

Certain rheological properties of the GO/SA ink are required for smooth printing. As shown in Figure 2f, the GO/SA ink displays typical shear‐thinning behaviors. The apparent viscosity of the GO/SA ink obviously drops along with the increase of the shear rates. Notably, the shear rate at the nozzle is usually orders of magnitude higher than that of the flow in other parts, indicating that the ink at the nozzle can be extruded.^26^ Besides, the GO/SA ink becomes viscous with increasing GO content. The addition of GO filler also enhances the storage elastic modulus of the ink (Figure S7a, Supporting Information). It should be noted that the loss modulus is larger than the storage elastic modulus of the printing ink (Figure S7b, Supporting Information), which indicates the extruded ink possesses a fluid‐like behavior. Thus, the fact that the confinement of substrate assisted by gravity enables the rearrangement of GO flakes on the substrate and the extruded filaments gradually collapse into anisotropic ribbons after drying can be understood. The printed feature is therefore not kept and these filaments merge into a fully infilled strip if the printing spacing is around the diameter of the extruded filaments. Apart from demanded rheological properties, their excellent interaction between GO filler and SA matrix enables a superior interfacial load transfer, and thus endows the printed strip with a fast shape‐morphing ability (see next).

### 2.3 Mechanism of Shape‐Morphing

The printed ribbons can response to multistimuli, including water (liquid or vapor), heat, and light, which can induce anisotropic expansion/contraction of the ribbons due to the anisotropic nacre‐like structure, which establishes the foundation of shape‐morphing behaviors of the printed GO/SA composite. As revealed by the analysis (Figure S3, Supporting Information), the selective arrangement of stacked GO flakes renders anisotropic deformation in the plane of the printed ribbons, and thus leads to different dimensional change ratio in their longitudinal (*S*_∥_) and vertical (*S*_⊥_) directions (Figure S8, Supporting Information). The absorbed water can lead to the expansion of the ribbons, whereas heat/light can result in contraction. Particularly, for morphing in liquid water, the printed strip should be cross‐linked with polyvalent metal ions (Ca^2+^ in this work) to fix its anisotropic microstructure and prevent dissolution (Figure S9a–d, Supporting Information). The functional groups of the SA matrix and GO flakes can easily react with most polyvalent metal ions to form cross‐linked networks against dissolution (Figure S9e,f, Supporting Information, and related discussion).^27^, ^28^

By controlling the printing pathway of the bottom and top layers in a vertically stacked bilayer, both of bent and twisted architectures can be produced using the printed strip when stimulated (as shown in Figure S10a,b and Movie S1, Supporting Information). For example, bending can be realized when the first layer is printed with an angle of 90° along the longitudinal direction of the strip and the second layer is printed with an angle of 0° (Figure S10a, Supporting Information). The bending stems from the anisotropic expansion/contraction of the printed ribbons in response to external stimuli, thereby inducing the mismatch of the two layers. Upon activation, the dominant in‐plane deformation of the first layer is along the long axis of the strip whereas that of the second one is transverse. Given that the two layers are vertically stacked and fixed, the deformation of the first layer along the longitudinal direction of the strip is constrained by the less deformed second layer, whereas the second one is passively forced to deform. The resultant bending moment induces the strip to bend, thereby reducing the stress between the midplane of the two layers. Alternatively, if the dominant deformation direction is at an angle along the longitudinal direction of the strip, the strip will twist upon stimulation. We showed an example of twisting by printing the first layer along an angle of 45° and the second layer along −45° (Figure S10b, Supporting Information).

The shape evolution of the printed strip can be understood according to the classical thermal expansion bilayer model proposed by Timoshenko.^29^ The resultant curvature can be expressed as the following equation (see details in Figure S11, Supporting Information, and its analysis)

(1) $$k=\frac{6{\left(1+m\right)}^{2}\cdot \Delta \alpha }{\left[3{\left(1+m\right)}^{2}+\left(1+mn\right)\left(m^{2}+1/mn\right)\right]\cdot h}$$

where *k* is the curvature of the resultant structure, *h* is the thickness of the strip, Δα is the mismatch in the dimensional change, *n* is the stiffness ratio of the two layers, and *m* is the thickness ratio of the two layers. Thus, control over the resultant morphing curvatures can be accomplished by adjusting intrinsic composite properties, including ink composition, GO alignment degree, crosslinking density of network, and geometrical configuration of the printed strip.

### 2.4 Intrinsic and Geometrical Control of Shape‐Morphing

**Figure 3:**
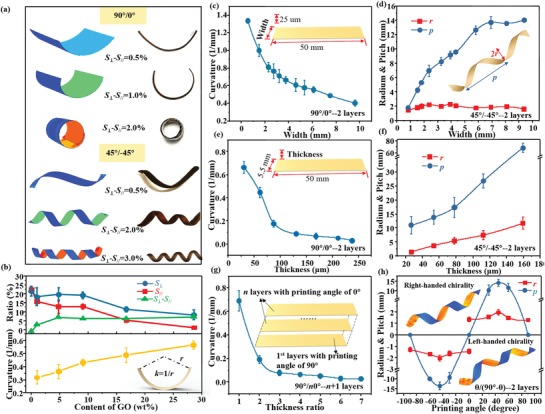
Controllable shape‐morphing of the printed composite. a) Experimental and simulation results showing bending and twisting modes of the printed strips with various values of *S*_⊥_–*S*_∥_ in water. b) Magnitude of *S*_⊥_, *S*_∥_, *S*_⊥_‐*S*_∥_ and resultant bending curvatures as functions of the GO solid contents. The inset illustrates the curvature of a bending strip. c) Resultant bending curvature as a function of widths of the strips printed in a 90°/0° configuration. The thickness is 25 µm and the length of 50 mm, as shown in the inset. d) Radii (*r*) and pitches (*p*) of resultant helixes as functions of widths of the strips printed in a 45°/−45° configuration. The thickness is 25 µm and the length is 50 mm. The inset depicts the *r* and *p* of a twisting strip. e) Bending curvature as a function of the thicknesses of the printed strips printed in a 45°/−45° configuration. The width is 5.5 mm and the length is 50 mm, as shown in the inset. f) *r* and *p* of the formed helixes as functions of thicknesses of the strips printed in a 45°/−45° configuration. The width is 5.5 mm and the length is 50 mm. g) The bending curvature as a function of layer number ratio (*n*) of multilayer of 0° printing angle to a layer of 90° printing angle as depicted in the inset. h) *r*, *p*, and chirality of the formed helixes as functions of printing angles of the strips. The inset shows simulation results of two helixes printed in 45°/−45° (form right‐handed chirality) and −45°/−135° (form left‐handed chirality) configurations.

The dimensional change discrepancy in vertical and longitudinal directions (*S*_⊥_−*S*_∥_) has a dominant effect on the extent of shape evolution of the printed strip. The simulation results shown in **Figure** **3**a prove that control over both bending and twisting modes can be realized by applying different magnitudes of *S*_⊥_−*S*_∥_. Along with the enlargement of the magnitude of *S*_⊥_−*S*_∥_, large curvatures of the resultant architectures can be obtained. As expected, experimental results are in good accordance with the simulation analysis.

Importantly, the content of GO in the GO/SA composite can influence the anisotropic dimensional change and the stiffness of the printed strip, and thus influence the extent of the shape evolution. As shown in Figure 3b, pure SA matrix shows a large isotropic swelling. By contrast, with increasing GO content, both magnitudes of *S*_⊥_ and *S*_∥_ decrease, which arises from the enhanced package of GO flakes in the SA matrix. Meanwhile, the deformation anisotropy (i.e., *S*_⊥_−*S*_∥_) also increases, thereby enhancing the resultant curvatures. As the GO content increases, the degree of confinement of anisotropically aligned GO flakes in the relatively reduced SA matrix becomes large, so the deformation anisotropy increases. Besides, the magnitude of *S*_⊥_−*S*_∥_ strongly depends on the extent of shear‐induced alignment of GO flakes by printing nozzles with different diameters (Figure S12, Supporting Information) and the cross‐linking density of the printed strip with Ca^2+^ ions (Figure S13, see details in the Supporting Information).

The extent of deformation can also be controlled by varying the geometrical configuration of the printed strips. For example, the resultant bending curvatures of the printed strips with a 90°/0° configuration decrease from about 1.4 to 0.4 mm^−1^ as the width increases from 1 to 10 mm (Figure 3c). As the width further increases, bending in the width direction which is vertical to the original bending plane appears and competes with the original bending, leading to the formation of a typical saddle‐like architecture. The pitch of the resultant helix for the printed strips with a 45°/−45° configuration increases with the width, whereas the radium slightly increases from about 1.2 to 2.0 mm and remains around 2.0 mm (Figure 3d). Besides, strips with a large thickness can be obtained by repeating the bilayer printing of 90°/0° layer by layer. With increasing thickness, the resultant curvature of 90°/0° strips sharply decreases (Figure 3e), which is in accordance with Timoshenko's model.^29^ The radium and pitch of the twisting helix for 45°/−45° strips increase as the thickness increases (Figure 3f). In addition, if a strip is printed with one layer in 90° and *n* layers in 0°, the resultant curvature of the strip will rapidly decrease with increasing *n* (Figure 3g).

The transition between bending and twisting mode was investigated to understand the programmability of the proposed 3D printing. By altering the printing angle from −90° to 90° in the first layer and maintaining the printing path of the second layer vertical to the first layer, we found that the radium and pitch of the resultant helix follow an opposite fluctuating trend. With the variation of the printing angle, the resultant structures transit between the bending mode and the twisting mode. For printing angles of −90°/−180°, 0°/−90°, and 90°/0°, the obtained strips are in bending mode, while for printing angles from −90°/−180° to 0°/−90° they are in twisting mode with a left‐handed chirality, and for printing angles from 0°/−90° to 90°/0°, they are in twisting mode with a right‐handed chirality (Figure 3h). Typical simulation results of 45°/−45° and −45°/45° strips are shown in the inset of Figure 3h. The above results prove that the morphology and the chirality of the obtained structures can be rationally tuned by adjusting the printing angles of the bilayers.

### 2.5 Complex Shape‐Morphing Architectures

**Figure 4:**
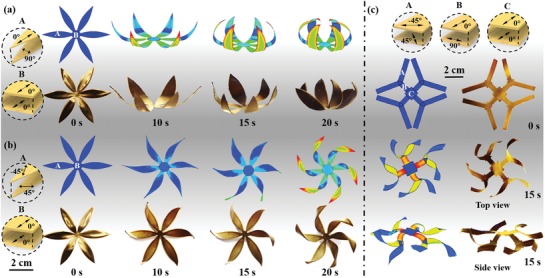
Shape evolution of rational designed planar structures into 3D architectures. a) Simulation and experimental results of a printed flower with six 90°/0° oriented petals and a 0°/0° oriented flower disc. b) Simulation and experimental results of a printed flower with six 45°/−45° oriented petals and a 0°/0° oriented flower disc. c) Simulation and experiment results of a complex structure composed of three parts with 45°/−45°, 90°/0°, and 0°/0° configuration, respectively.

On the basis of the programmable morphing modes of the printed strips mentioned above, versatile 3D architectures can be realized by the 3D printing technique. For demonstration, we printed a planar flower with six 90°/0° oriented petals and a 0°/0° oriented central disc. As shown in **Figure** **4**a, the petals gradually close in about 20 s, which is in good consistency with the simulation results. As indicated by the simulation results, the bending of petals is induced by the large stress generated in the bilayer, whereas the central disc remains flat because of free of stress. By contrast, a clockwise twisting configuration of petals can be obtained by printing 45°/−45° oriented bilayer petals (Figure 4b), which is also in good consistency with the simulation results.

Figure 4c shows a more complex architecture fabricated by the programmable 3D printing technique. We printed a 2D structure composed of three parts, including 45°/−45° oriented exterior (A) and a 0°/0° oriented center (C), which are connected by 90°/0° oriented joints (B). As shown in Figure 4c, it transforms into an intricate 3D shape with twisting exterior (A) and a planar panel (C) joined by an out‐of‐plane bending joint (B). The as‐formed 3D structure matched well with the predicted structure by simulation. Our results indicate that the proposed printing technique based on the GO/SA composite, along with the simulation approach, is a reliable approach for fabricating versatile intricate 3D architectures, which is beyond the capability of conventional fabrication methods.

### 2.6 Multistimuli‐Responsive and Reversible Fast Shape‐Morphing

**Figure 5:**
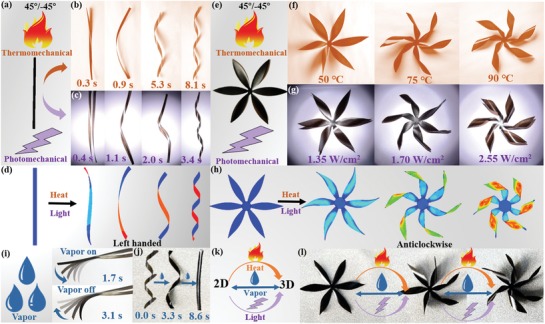
Multistimuli responsivity of the printed composite. a–c) Thermomechanical and photomechanical response of a 45°/−45° oriented strip. The snapshots show its twisting response to heat (50 °C) (b) and to light (xenon lamp, 1.35 W cm^−2^) (c). d) Simulation snapshots of twisting evolution of a 45°/−45° oriented strip. e–g) Thermomechanical and photomechanical response of a 45°/−45° oriented flower. f,g) The snapshots show its twisting response to heat and to light. h) Simulation results of a 45°/−45° oriented flower with various magnitude of anisotropic contraction in the bilayer. i) Deformation and recovery of a 45°/−45° oriented strip under vapor stimulus. j) Recovery of a helix under vapor stimulus. k) Reversible shape‐morphing of the printed composite under vapor stimulus or combination of multistimuli (vapor, heat, and light). l) Reversible shape‐morphing of a flower under stimuli of light, heat, and vapor.

Remarkably, the printed composite shows a fast shape‐morphing response to multistimuli, including heat, light, and water (**Figure** **5** and Movies S2 and S3, Supporting Information). As demonstrated in Figure 5a,b, a 45°/−45° oriented strip is heated at a temperature of 50 °C and the strip transforms to a helix in ≈8.1 s. Similarly, a 45°/−45° oriented strip shows a fast photomechanical response to light and rapidly turns to a helix with left‐handed chirality within ≈3.4 s (Figure 5a,c). The morphing response toward heat and light can be ascribed to the anisotropic shrinkage of the printed ribbons and thereby the anisotropic contraction of the vertically stacked bilayers. Water molecules in the printed ribbons were driven out at high temperatures, leading to anisotropic shrinkage of the ribbon. As evidenced by the thermogravimetric analysis (Figure S14, Supporting Information), the water content in the as‐printed ribbon is about 9.47 wt% and drops to 6.34 wt% after being heated at 50 °C. Light can elevate the temperature of the printed composite due to the solar thermal conversion of GO flakes in the SA matrix. The analysis was confirmed by the simulation results (Figure 5d), which show that a 45°/−45° oriented strip undergoes a helical transformation with a left‐handed chirality when the printed bilayer anisotropically shrinks with increasing temperature. By contrast, the helix with a right‐handed chirality can be obtained in water as previously demonstrated, owing to a reverse deformation (i.e., swelling) of the printed ribbon after water absorption.

In addition, the extent of shape evolution of a flower (with six 45°/−45° oriented petals) in response to various temperature and light intensity (Figure 5e and Movie S4, Supporting Information) was investigated. Upon heating at a temperature, the planar flower petals turn to an anticlockwise twisting configuration and retain their shape after reaching an equilibrium (Figure 5f). With increasing temperature, the extent of twisting enhances, which can be ascribed to the increased shrinkage of the anisotropic bilayer. This phenomenon is consistent with the behavior of the printed flower under different light intensity (Figure 5g). As suggested by the simulation results (Figure 5h), the extent of the twisting after reaching the equilibrium state depends on the magnitude of anisotropic shrinkage, which can be tuned by altering heating temperatures or light intensity.

Notably, reversible shape‐morphing of the printed composite can be realized when stimulated by water vapor (Figure 5i). When vapor is applied, a 90°/0° oriented strip quickly bends to an equilibrium state in 1.7 s and returns to its original shape in 3.1 s when the vapor is turned off. The fast reversible shape‐morphing behaviors upon stimuli of water vapor can be ascribed to the fast adsorption and desorption of water molecules in the printed composite, which can induce fast and reversible anisotropic deformation of the strip. Besides, the mechanical properties of the printed composite were measured under different relative humidity at 70 °C (Figure S15, Supporting Information). More water molecules are absorbed in the composite under higher humidity, leading to decreased elastic modulus and enhanced elongation at break. In addition, we investigated the repeatability of the printed composite for shape morphing by cyclic water vapor treatment for more than 100 times, demonstrating its good stability (Movie S5, Supporting Information). Similarly, the deformation induced by heat and light can also be reversed by using water vapor. As shown in Figure 5j, a helix (triggered by heat) returned to its original shape in only 8.6 s by vapor activation. Based on this, reversible shape‐morphing can be realized by using vapor or combining different stimuli (Figure 5k). Figure 5l shows an example, where reversible shape‐morphing of a flower (with six 45°/−45° oriented petals) is stimulated using heat, light, and water vapor (Movie S6, Supporting Information).

The printed composite shows a fast response time (several seconds) to multistimuli, including heat, light, and water, which is superior to most of the reported shape‐morphing materials (Figure S16, Supporting Information), especially shape‐morphing hydrogels (the composite containing GO additive in SA matrix can be regarded as hydrogel when swelling and shape‐morphing in water).^8^, ^30^, ^31^, ^32^, ^33^, ^34^, ^35^, ^36^, ^37^, ^38^, ^39^, ^40^, ^41^, ^42^, ^43^ The reason can be explained as the following. Two most important factors that determine the response speed of actuation are mass transport efficiency of water and toughness of the hydrogels.^44^, ^45^ Our printed hydrogel composite has a thin bilayer structure (with a thickness of dozens of micrometers after filaments collapsing and drying), which favors the fast water transportation. Besides, the stiffness of our printed hydrogel composite is as high as 7.4 GPa, which is several orders of magnitude higher than that of most soft hydrogels with long response time from dozens of minutes to several hours. Based on its superior performance, the printed composite can be used as a stimuli‐responsive actuator for soft robotics. For demonstration, we fabricated a vapor‐responsive gripper, which can grasp and release items upon on vapor on/off stimuli (Figure S17, Supporting Information, and related discussion). Besides, the printed hydrogel composite can also be employed as an efficient and visualized indicator to qualitatively detect the concentration of heavy metal ions in polluted water (Figure S18, Supporting Information, and related discussion).

## 3 Discussion

In summary, we report the direct formation of spontaneously aligned GO in SA hydrogel with locally controlled texture via a direct‐ink‐writing process, which can transform into various 3D architectures upon multistimulation. The GO flakes are highly aligned because of the large shear force at the printing nozzle, leading to the formation of a brick‐and‐mortar like structure that shows anisotropic response to multistimuli. By tuning the printing pathway of each layer in vertically stacked bilayer structures, morphing modes of bending and twisting can be realized. Various stimuli, including heat, light, and water, can induce the fast shape‐morphing of the printed structures. Directed by finite element simulation, versatile architectures were obtained by synergistically manipulating the morphing modes through designing the printing pathway. Particularly, reversible actuation can be achieved by using water vapor as one of the stimuli. Based on the shape‐morphing of the GO/SA composite, a stimuli‐responsive gripper for soft robotics was demonstrated. In general, our shape‐morphing hydrogel by the 3D printing method shows superiority in several aspects, including its benign components, easy and fast production, capability for printing arbitrary shapes, fast response, and multistimuli responsiveness. Besides, this direct‐ink‐writing method may also be applicable for fabricating other aligned 2D flakes in suitable polymer matrixes, as long as the components have compatible interfaces and the ink has suitable rheological properties for 3D printing. We foresee that the printed multistimuli‐responsive composite with a reversible and fast response will pave an avenue for the development of a variety of smart systems in the future.

## 4 Experimental Section

##### Preparation of GO/SA Composite Ink

GO was synthesized according to the modified Hummers' method.^46^ A series of GO/SA composites ink with various GO solid contents (weight ratio) was obtained by adding a different amount of GO into 1000 mg SA and 12 mL deionized water. Unless otherwise stated, the amount of GO in the composite used in this work is 200 mg. The mixture was mildly stirred using a mechanical mixer at 100 rpm for 5 min and then formed a uniform paste using a speed mixer at 3500 rpm for 10 min.

##### Characterization of GO/SA Composite

The topography of GO flake and GO/SA composite was characterized using an AFM (Oxford Instruments Asylum Research, Inc.). FTIR spectra were obtained using a Nicolet 6800 FTIR spectrometer. XRD spectra of GO/SA with various GO contents were carried out using a laser Al Kα radiation (Thermo Scientific Escalab 250Xi). The high alignment of GO in printed ribbons was evidenced by 2D‐WAXD pattern (Bruker D8). The rheological properties of GO/SA composite ink were characterized by a rheometer (Anton Paar, MCR 301). A constant frequency of 1 Hz with a stress sweep from 10^−1^ to 10^3^ Pa was set to obtain the storage modulus and loss modulus.

##### Printing of GO/SA Composite

GO/SA ink in an injection syringe was extruded by a syringe pump (Lead Fluid, TSD01) and connected to a nozzle (150–1000 µm) fixed on a 3D printer (Anycubic I3 MEGA). Various configurations of 3D printed sheets were designed with commercial software (3ds max) and converted to G‐codes that determine the printing pathways by another commercial software (simplify 3D). The ink was printed on a polyethylene terephthalate film and left to dry until it could be peeled off for characterization or further usage.

##### Characterization of the Printed GO/SA Composite

The morphology of the printed GO/SA composite ribbon was obtained using a scanning electron microscope (Zeiss, Merlin) and an optical microscope (Leica, DMi8 S). The anisotropic mechanical properties of the printed strips with a gauge length of 10 mm and a width of 5 mm were mounted on a paper frame by gluing the ends of strips to the frame and measured at a tensile rate of 0.5 mm min^−1^ using a tensile tester (Shimadzu, AGS‐X). Particularly, in order to avoid dissolution of the structures in water, the 3D printed GO/SA composite was cross‐linked with Ca^2+^ by immersing in 0.5 m CaCl_2_ aqueous solution for different time, and then immersed in water for the investigation of shape‐morphing behavior in water. Unless otherwise stated, investigation of shape‐morphing behavior of the printed composite was conducted in water. For treatment with heat, light, and water vapor, the cross‐linking of the printed composite with metal ions is not needed. The formation of water vapor was realized using a humidity chamber (Eyela, KCL‐2000). The treatment of heat was realized using a hot plate (Wiggens, WH200D). The light source used is a xenon light (Ceaulight, CEL‐HXF300). Since the printed composites are responsive to water vapor, an environment with high humidity and temperature may influence their performance. Unless otherwise stated, the fabrication, storage, and test process of the printed composite were conducted in the ambient environment (at a temperature of 25 °C and 20% relative humidity).

##### Finite Element Analysis

To get the simulation results shown in Figures 3, 4, 5, a commercial finite element analysis software ABAQUS was used and vertically stacked anisotropic bilayer with fixed midplane was employed. The Poisson's ratio of the printed strip is set to be 0.30. The elastic moduli along the longitudinal (printing direction) and transverse orientation were set as 7.4 and 2.6 GPa, which was set according to the experiment results. To predict the deformation of various structures, the orientations of the elastic moduli and the anisotropic deformation ratios can be easily tuned.

For the computational fluid dynamics simulation shown in Figures S2 and S3 (Supporting Information), the GO/SA ink follows a typical power viscosity model (Figure 2f), which is fitted and applied into simulation. A tapered printing nozzle has an inlet with a diameter of 4 mm, and an outlet with a diameter of 150 µm. The ink was extruded with a speed of 30 mm s^−1^. The simulations were realized using COMSOL Multiphysics software.

## Conflict of Interest

The authors declare no conflict of interest.

## Author Contributions

Y.Y.Z. supervised the project. M.C.Z. designed and performed the experiment. Y.L.W., M.Q.J., C.Y.W., and X.P.L. participated in part of the experiment. All the authors discussed the results. M.C.Z. and Y.Y.Z. co‐wrote the manuscript with feedback from all authors.

## Supporting information

Supporting Information

Supplemental Movie 1

Supplemental Movie 2

Supplemental Movie 3

Supplemental Movie 4

Supplemental Movie 5

Supplemental Movie 6
