# Supplementary material for: Spontaneous Alignment of Graphene Oxide in Hydrogel during 3D Printing for Multistimuli‐Responsive Actuation
Source: Adv Sci (Weinh). 2020 Jan 30;7(6):1903048. doi: 10.1002/advs.201903048 (PMC7080512; doi:10.1002/advs.201903048)
Supplement: Supplementary file 1 — Supporting Information [file ADVS-7-1903048-s001.pdf]

Copyright WILEY-VCH Verlag GmbH & Co. KGaA, 69469 Weinheim, Germany, 2020.

ADVANCED SCIENCE
Open Access

Supporting Information

for *Adv. Sci.,* DOI: 10.1002/advs.201903048

Spontaneous Alignment of Graphene Oxide in Hydrogel during 3D Printing for Multistimuli-Responsive Actuation

*Mingchao Zhang, Yiliang Wang, Muqiang Jian, Chunya Wang, Xiaoping Liang, Jiali Niu, and Yingying Zhang**

Copyright WILEY-VCH Verlag GmbH & Co. KGaA, 69469 Weinheim, Germany,

2020.

Supporting Information

Spontaneous alignment of graphene oxide in hydrogel during 3D printing for multi-stimuli-responsive actuation

Mingchao Zhang, Yiliang Wang, Muqiang Jian, Chunya Wang, Xiaoping Liang, Jiali Niu and Yingying zhang*

The supplementary file includes:

- Supplementary Figure S1 to S18
- Supplementary Video S1 to S6.
- Supplementary Discussions related to Figure S2-S4, S9, S11-S13, and S17-S18.

The sequence is the same as they are mentioned in the main text.

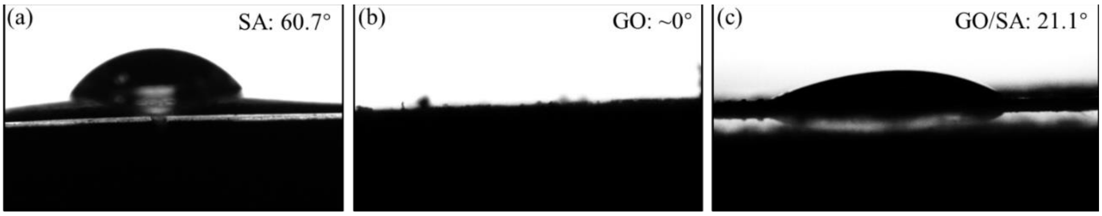

Figure S1. (a-c) Water contact angle of SA (60.7°) (a), GO (~0°) (b), and GO/SA film (21.1°) (c).

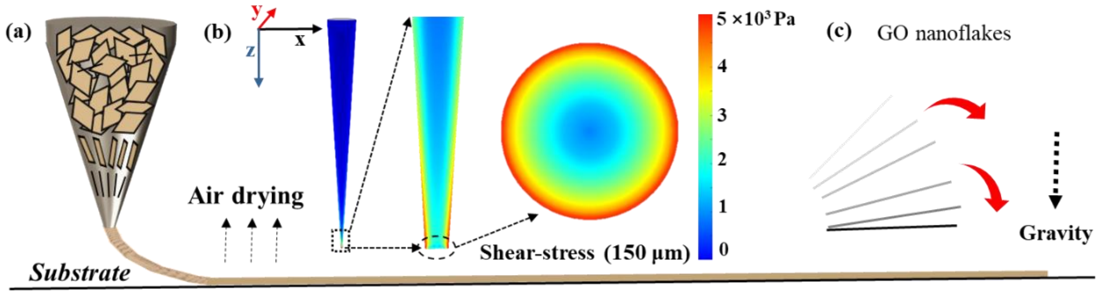

Figure S2. The mechanism for the spontaneously alignment of GO flakes in SA matrix during the printing and drying process. (a) Schematic illustration showing the alignment of GO flakes along the longitudinal direction of the filament during the extruding and drying. (b) Distribution of calculated shear-stress in GO/SA ink induced by a tapered printing nozzle. (c) Schematic illustration showing the GO flakes tends to lie flat on the substrate as a result of gravity effect.

The orientation of GO in the SA matrix during printing and drying process was systematically investigated. As the evolution of GO orientation shown in Figure S2a, the GO/SA printing ink is extruded from a tapered printing nozzle, and the extruded filament is then deposited on a substrate and left to dry. As the theoretical results shown in Figure S2b, the printing ink will undertake incremental shear-stress when extruded from a tapered printing nozzle, which renders these GO flakes to parallelly align along the direction of ink flow. After the ink is printed on the substrate, the

aligned GO flakes tend to lie flat as a result of the confinement of substrate and gravity effect (Figure S2c). Therefore, the printed GO flakes in the SA matrix will be horizontally stacked after drying, forming a bricks-and-mortar microstructure.

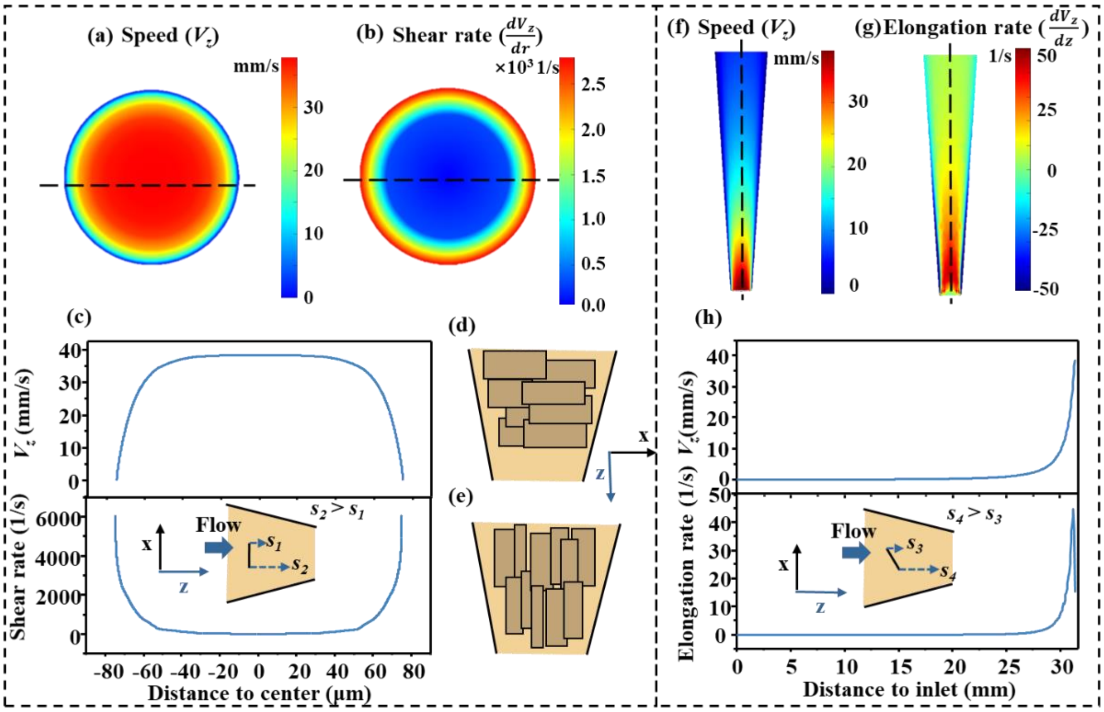

Figure S3. Analysis on the orientation of GO flakes in a SA matrix when extruded from a tapered nozzle using CFD simulation. (a) Distribution of flow speed at the nozzle outlet. (b) Distribution of shear rate at the nozzle outlet. (c) Extracted flow speed and shear rate along the diameter of the nozzle outlet. Inset shows the stress difference loaded on the different part of a vertically aligned GO flake. (d-e) Orientation scenarios of GO flakes with short side (d) and long side (e) along the flow direction when extruded from the nozzle outlet. (f) Distribution of flow speed in the nozzle. (g) Distribution of elongation rate in the nozzle. (h) Extracted flow speed and elongation rate along the axis of the nozzle. Inset illustrates that the existence of elongation rate promotes orientation of GO flakes.

By the aid of computational fluid dynamics (CFD) simulation, the orientation of GO flakes in SA matrix when extruded from a printing nozzle was analyzed. The dynamic viscosity behavior of GO/SA ink follows a typical power viscosity model (Figure 2f), which is fitted and applied into the simulation. Extracted from the flow speed (V_z) distribution at the nozzle outlet (Figure S3a), shear rate ($\frac{dV_z}{dr}$) along its diameter can be calculated (Figure S3b). The values of shear rate show a U shape distribution along the diameter of the printing nozzle, where it significantly increases when away from the center of the nozzle (Figure S3c). The drastically increased shear rate forces the GO flakes to parallelly align along the flow direction. As schematically shown in the inset of Figure S3c, randomly aligned GO flakes bear unbalanced shear force, which causes large resistance for the ink flow. Thus these GO flakes are forced to roll over until being parallelly aligned to the flow direction to minimize the flow resistance.

It is should be noted that a parallelly aligned GO flake with its long side perpendicular to the flow direction bears larger shear stress than that with its short one, leading to its deflection towards another direction (Figure S3d). Therefore, the GO flakes tend to be aligned with its long sides parallel to the flow direction (Figure S3e). Besides, the employed tapered printing nozzle significantly accelerates the flow speed of ink as the channel gradually becomes narrow (Figure S3f), generating an elongation rate ($\frac{dV_z}{dr}$) that drastically increase when the ink is flowing through the nozzle tip (Figure S3g). The positive elongation rate further improves the GO orientation by forcing randomly distributed GO flakes parallelly aligned along the flow direction (Figure S3h). The

alignment of GO flakes in the extruded filament endows the printed strips with anisotropic mechanical properties.

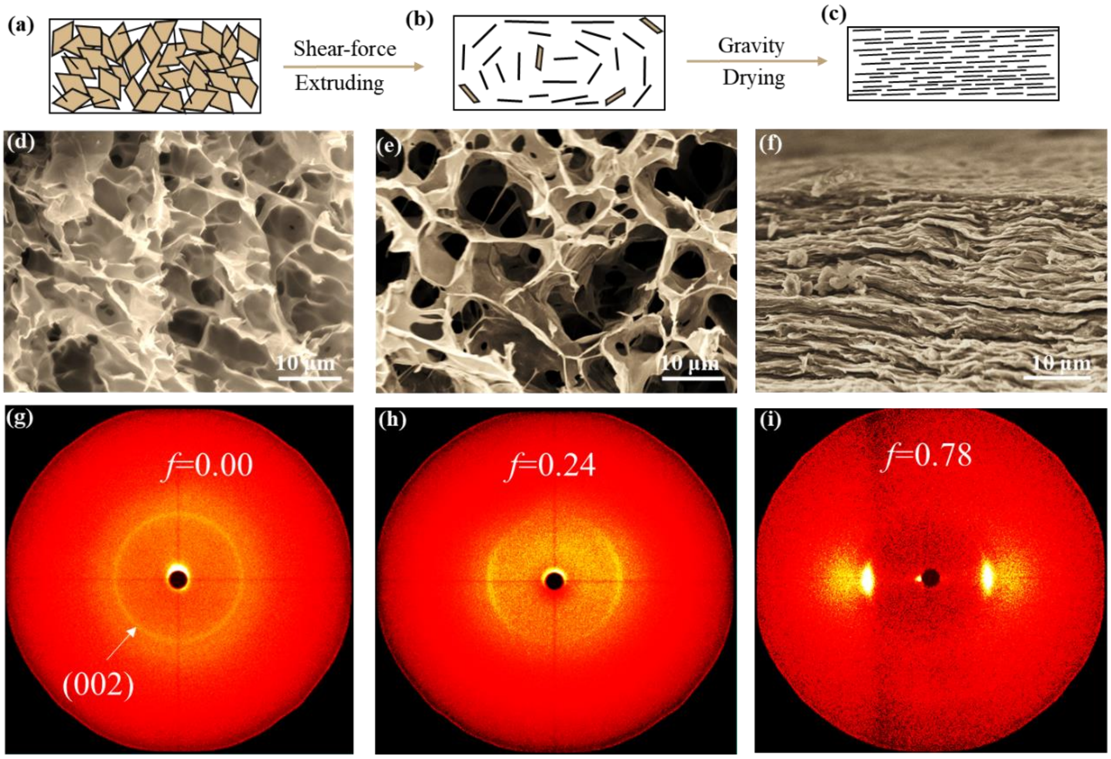

Figure S4. Illustration and experimental results showing the evolution of GO orientation in the SA matrix during the printing process. (a-c) Schematic illustration showing the evolution of the alignment of GO flakes. When extruded from the printing nozzle, the flakes are aligned parallelly along the filament (b) and gradually lie flat on the substrate after drying (c). (d-f) SEM image of freeze-dried GO/SA printing ink (d), the cross-section of freeze-dried extruded filament (e), and the cross-section of dried ribbon (f). (g-i) 2D WAXD patterns of GO/SA printing ink (g), extruded filament (h), and dried ribbon (i).

As illustrated in Figure S4a-S4c, the orientation degree of the GO flakes gradually increases during the extruding and drying process, which can be evidenced by the 2D-WAXD patterns and the derived Herman's orientation factor (f). It is noted that the value of f reaches 1 when GO flakes are perfectly aligned and 0 indicates GO flakes are in completely random orientation. The GO/SA printing ink shows an

isotropic nature with randomly distributed GO flakes in SA matrix (Figure S4d), which can be evidenced by the typical diffraction ring pattern of the 2D-WAXD and its corresponding value of f=0 (Figure S4g). When it flows through a narrow and tapered nozzle, the GO flakes are then parallelly aligned along the direction of extruded filament (Figure S4e), which is proved by the increased value of f =0.24 (Figure S4h). After the extruded filament is printed on a substrate, the GO orientation further enhances as these GO flakes are eventually aligned horizontally as the result of substrate confinement and gravity effect (Figure S4f), which can be revealed by the further increased value of f=0.78 (Figure S4i). In summary, the shear force in the extruding process and the gravity effect during the drying process endow the resultant strip with highly anisotropic properties.

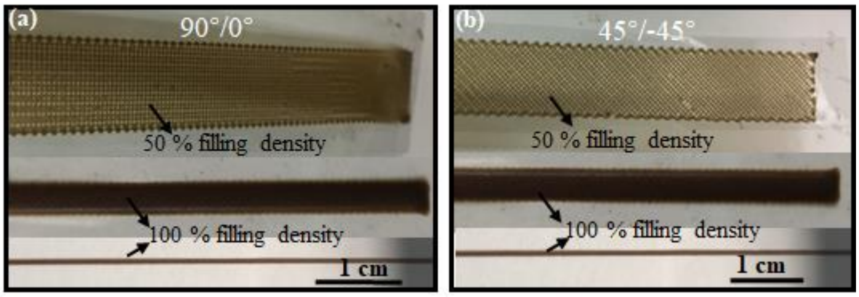

Figure S5. Photographs of printed trips with different filling densities and dimensions. (a) Printed strips with a 90°/0° bilayer configuration. (b) Printed strips with a 45°/-45° bilayer configuration.

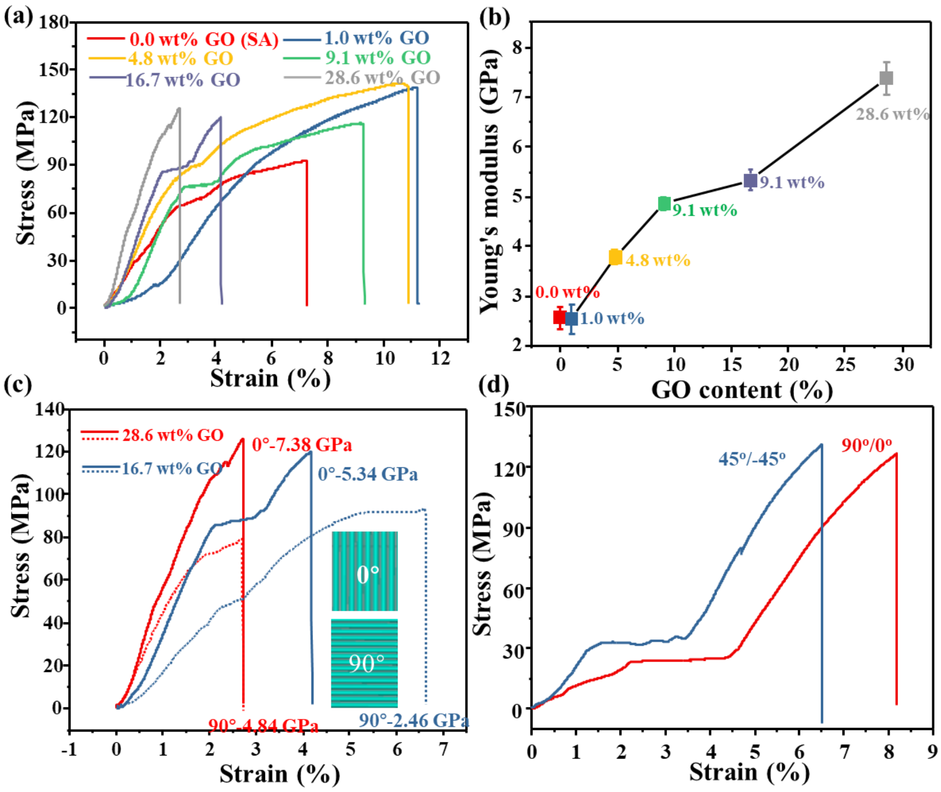

Figure S6. Mechanical properties of the printed strips. (a) Stress-strain curves along the printed direction of the printed strips with different GO solid content. (b) Young's modulus as a function of GO contents. (c) Mechanical properties along longitudinal (0°) and vertical (90°) direction of the two typical printed strips. Insets are the schematics of the printed strips with longitudinal directions along (0°) and transverse (90°) to the printing pathways, respectively. (d) Stress-strain curves of printed strips with lattice configurations of 90°/0° and 45°/−45°, respectively.

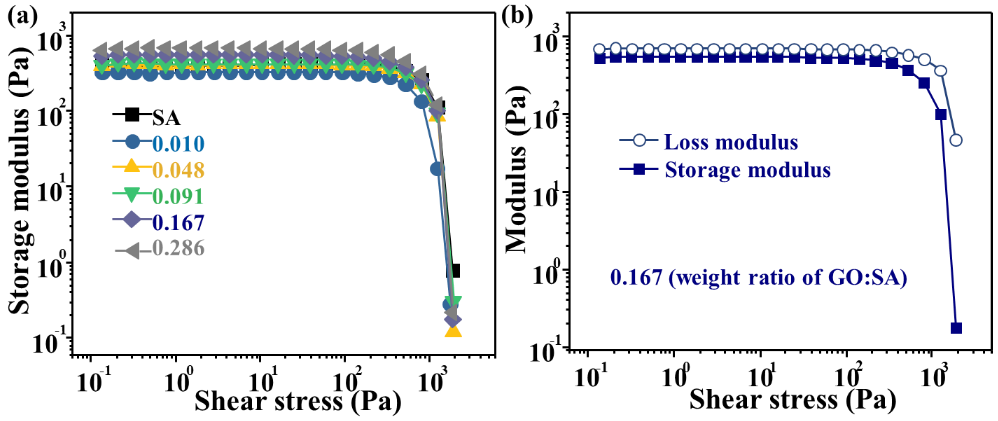

Figure S7. (a) Storage modulus of GO/SA inks with various GO solid content as a function of shear stress. (b) Storage and loss modulus of GO/SA ink with GO content of a weight ratio of 0.167 as a function of shear stress.

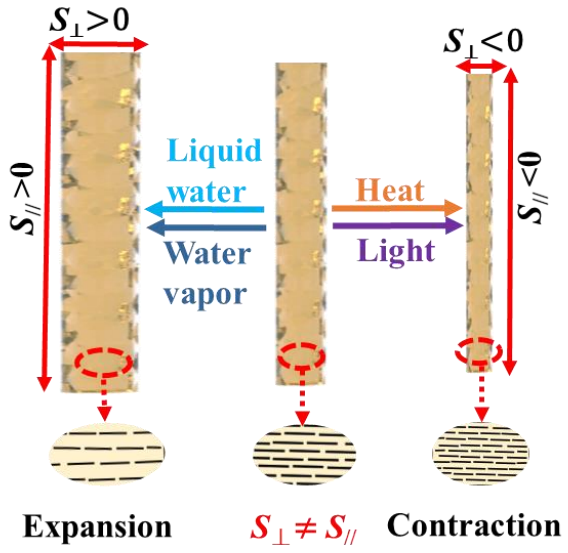

Figure S8. The anisotropic expansion/contraction of a printed ribbon under various stimuli.

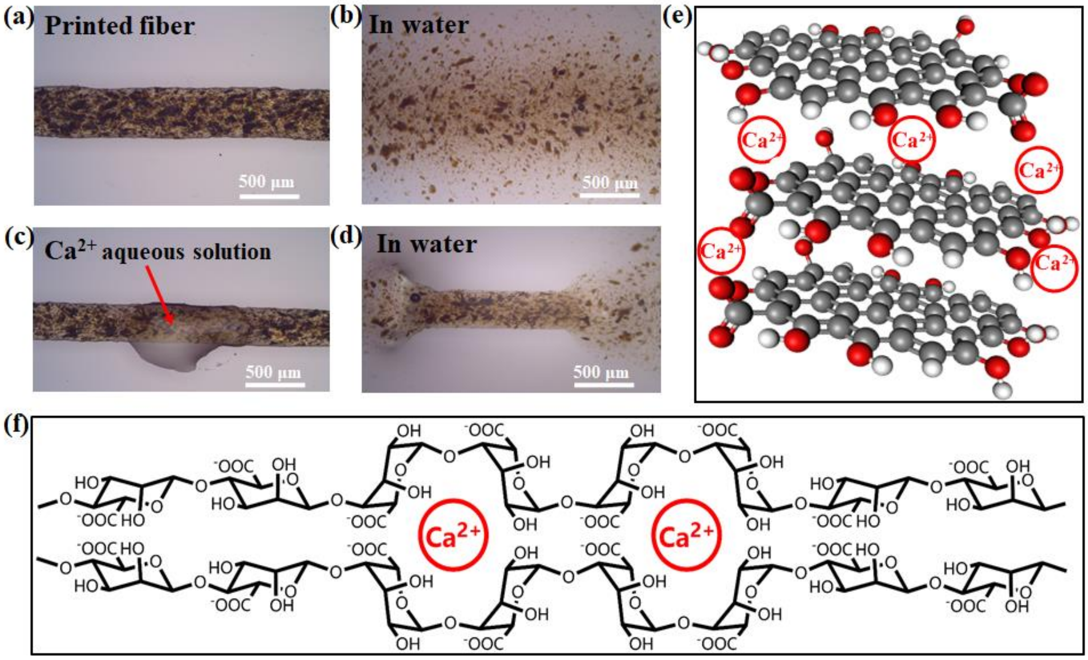

Figure S9. The cross-linking of GO/SA composite with Ca^{2+} ions. (a) Optical image of a printed ribbon. (b) Optical image of the dissolved ribbon after immersed in water. (c) Optical image of a partially cross-linked ribbon with Ca^{2+} ions. (d) Optical image of the remaining ribbon in water after cross-linked with Ca^{2+}. (e) Schematic of GO cross-linked with Ca^{2+} ions. (f) Schematic of SA cross-linked with Ca^{2+}.

It should be noted that our printed composite should be cross-linked with polyvalent metal ions to prevent its dissolution in water. As shown in Figure S9a and S9b, if the printed fiber was immersed in water, it could not maintain its original shape and quickly dissolved into water. In contrast, when cross-linking with polyvalent metal ions ($CaCl_2$ solution), the cross-linked part remained their fiber shape when immersed in water, while other parts without cross-linking with Ca^{2+} dissolved into water (Figure S9c and S9d).

As our printed structure is composed of aligned GO in SA matrix, both of the two components are readily to cross-link with Ca^{2+}, and form a tight network to prevent their dissolution in water. As schematically illustrated in Figure S9e, wide range of oxygen-containing function groups on the basal

planes and edges of GO enable the binding of Ca^{2+}, thus forming ionic bridges among the neighboring flakes. Besides, SA is a natural and biocompatible anionic polysaccharide copolymer, which possesses repeating units with oxygen-containing functional groups (-O-, -OH, and –COO−). The blocks of guluronic units cooperatively interact with Ca^{2+}, and form a three-dimensional linkage among different chains, where the Ca^{2+} ions were embedded in a way like eggs in a cardboard box (Figure S9f). These two components readily interact with Ca^{2+} and interlock each other, forming a complex cross-linked network, which prevents their dissolution in water and only allows anisotropic swelling of the network.

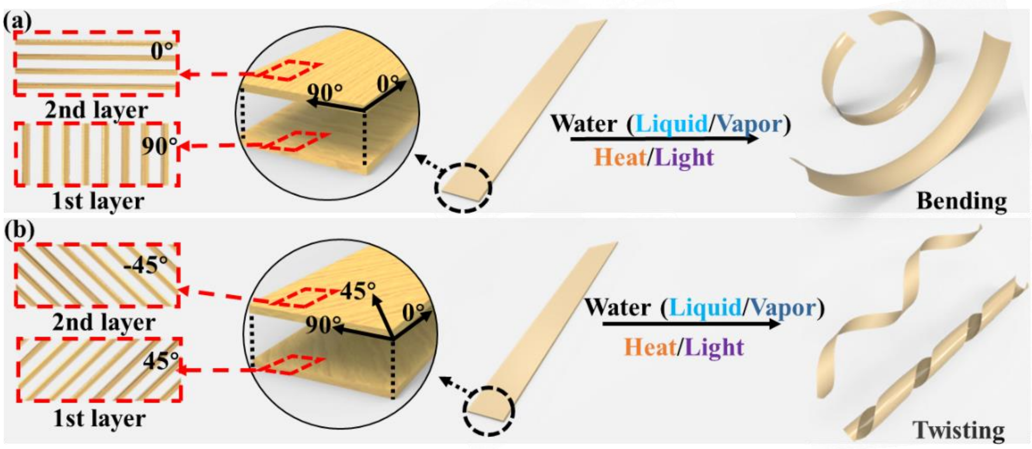

Figure S10. (a) Schematics of 90°/0° bilayer strip and its bending under various stimuli. (b) Schematics of a 45°/-45° bilayer strip and its twisting under various stimuli.

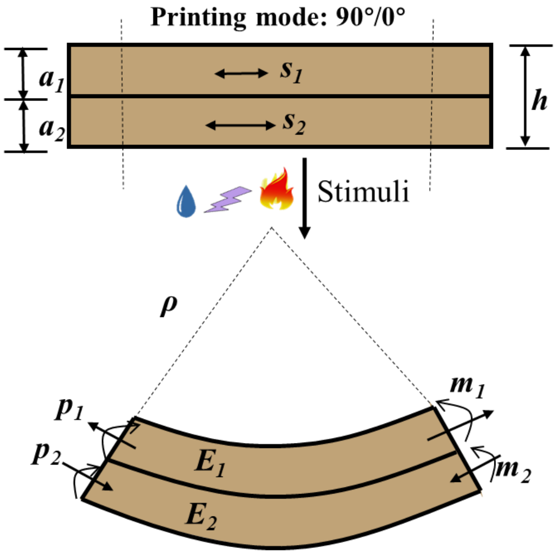

Figure S11. Bending model of a 90°/0° oriented strip.

Figure S11 shows a typical bending model. We assume that the cross-sections of the original printed strip always remain plane during the bending process (pure bending). A part is taken out from the strip for analysis. The bending stems from the anisotropic expansion/contraction of the printed ribbons in response to external stimuli, thereby inducing the mismatch of the two layers. Let's consider if $S_1 < S_2$, the bending direction will be convex down, where S_1 and S_2 are the dimensional change ratio (strain) along the longitudinal direction of the two layers, which is also equal to the strain in the longitudinal ($S_{//}$) and vertical ($S_{\perp}$) directions of the printed ribbon (i.e. $S_1 - S_2 = S_{\perp} - S_{//}$). For the printed two layers, because the two layers are vertically stacked and fixed, the deformation of the bottom layer along the longitudinal direction of the strip is constrained by the less deformed top layer, whereas the top one is passively forced to deform. The generated internal compressive force (p_2)/bending moment (m_2) and tensile force (p_1)/bending moment (m_1) thus act on the cross-section of the bottom

and top layer, respectively. Since there exist no external forces acting on the strips, the strip remains in equilibrium, and it follows:

$$P_1 = P_2 \tag{1}$$

$$\frac{P_1 h}{2} = m_1 + m_2 \tag{2}$$

The bending moment can also be obtained by:

$$m_1 = \frac{E_1 I_1}{\rho} \tag{3}$$

$$m_2 = \frac{E_2 I_2}{\rho} \tag{4}$$

Where E_1 and E_2 are Young's modulus of top and bottom layer, which can be measured by tensile test (Figure S6). I_1 and I_2 represent the moment of inertia of the top and bottom cross-section, calculated about the neural axis. ρ is the radius of curvature of the strip.

Because the two layers are fixed, the strains in the interface of both layers must be the same. Thus we can obtain:

$$\frac{P_1}{E_1 a_1 w} + s_1 + \frac{a_1}{2\rho} = \frac{P_2}{E_2 a_2 w} - s_2 - \frac{a_2}{2\rho} \tag{5}$$

Where w is the width of the strip, and here we take it as unity length. By using (1) ~ (5), we obtained :

$$\frac{1}{\rho} = \frac{6(1+m)^2 \cdot \Delta\alpha}{[3(1+m)^2 + (1+mn)(m^2 + 1/mn)] \cdot h} \tag{6}$$

Where $\Delta\alpha = S_1 - S_2 = S_{\perp} - S_{//}$, where $m = a_1/a_2$, $n = E_1/E_2$.

Noted that the bending model can be also applied to the cross-section area. The mismatch in this direction renders the cross-section convex up, thus forming a saddle-like architecture. Since the width of the strip is much smaller than its length, the bending of the cross-section is not apparent. Besides, if the dominant deformation is not along the longitudinal direction of the strip (enabled by changing the printing angles), a twisting architecture will be obtained, which shares similar analysis mentioned above.

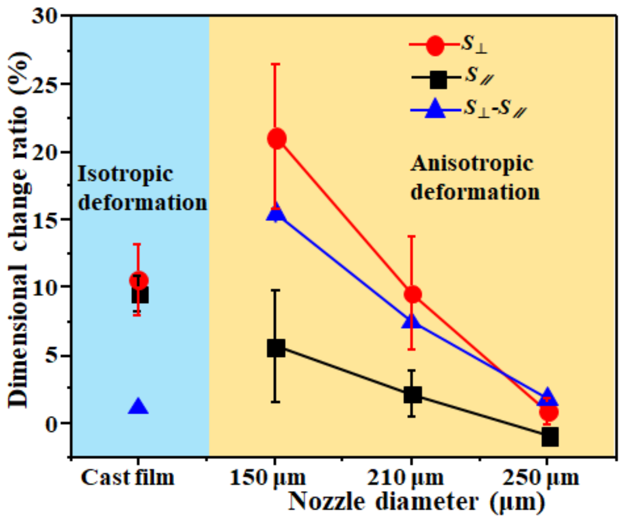

Figure S12. Magnitude of $S_{\perp}$, $S_{//}$, and $S_{\perp}$-$S_{//}$ of cast film and printed films obtained by using printing nozzles with various diameters.

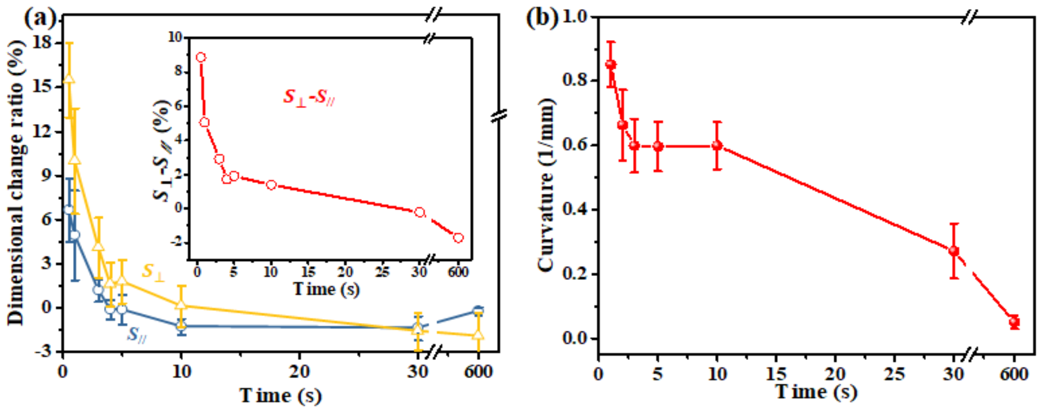

Figure S13. (a) Magnitude of $S_\perp$, $S_{//}$, and $S_\perp$-$S_{//}$ (in the inset) as functions of cross-linking time of the printed strips with Ca^{2+} ions. (b) The resultant bending curvature as a function of cross-linking time of the 90°/0° oriented strips.

Besides, the magnitude of $S_\perp$-$S_{//}$ strongly depends on the extent of shear-induced alignment of GO flakes. The alignment of GO flakes, and hence the deformation anisotropy can be tuned by the nozzle diameter. As shown in Figure S12, the magnitude of $S_\perp$-$S_{//}$ raises as the decrease of the nozzle diameter, which indicates that a small nozzle diameter leads to a large shear force and high extent of alignment of GO flakes. In shape contrast, the cast film shows an isotropic dimensional change in the plane of strips. In addition, the crosslinking density of the printed strips with Ca^{2+} also determines the morphing degree. When cross-linked in 0.5 M Ca^{2+} aqueous solution, the printed strips show a decreased magnitude of $S_\perp$, $S_{//}$ and even $S_\perp$-$S_{//}$ (Figure S13a) when increasing cross-linking time, which can be ascribed to the fact that a longer cross-linking time, and hence a denser cross-linked network leads to a decreased water absorption and thus dimension change. The corresponding resultant bending curvatures decrease with the increase of the cross-linking time shown in Figure S13b.

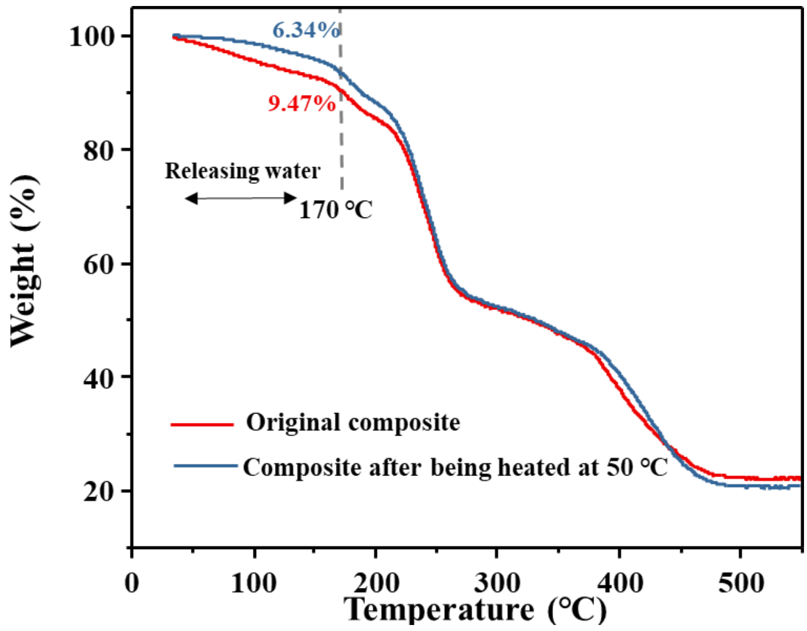

Figure S14. Thermogravimetric analysis of the printed composites before and after being heated at 50 ℃.

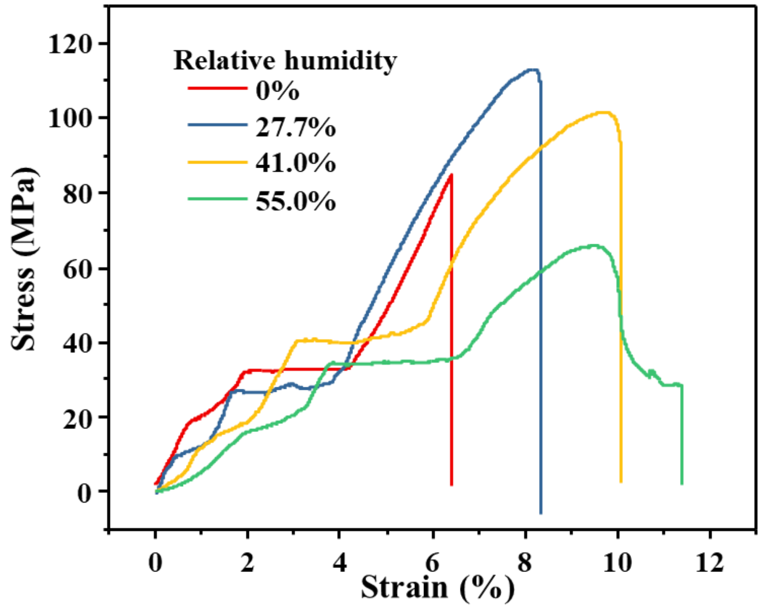

Figure S15. Mechanical properties of the printed composites under different relative humidity at 70 ℃.

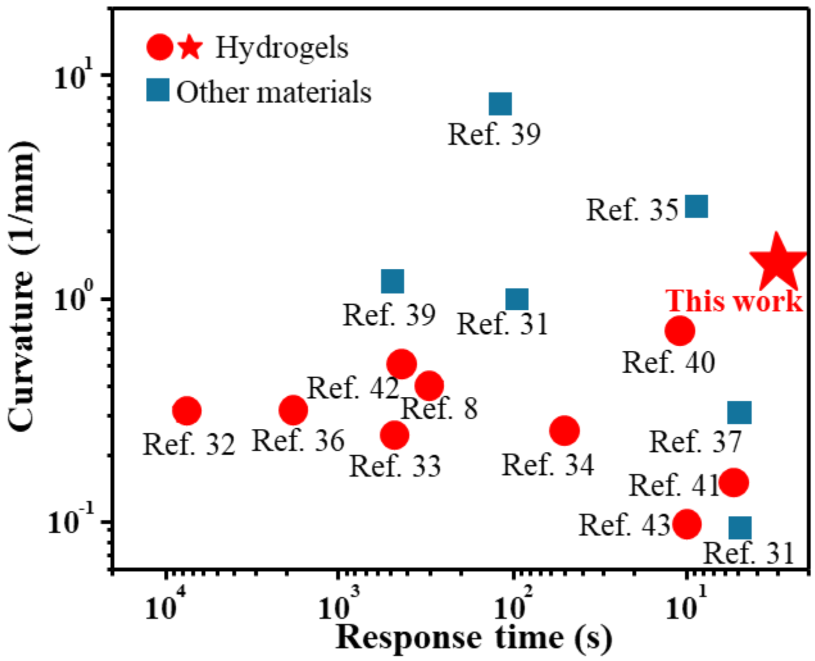

Figure S16. Response time and resultant curvature of the printed hydrogel composite compared with other reported shape-morphing materials.

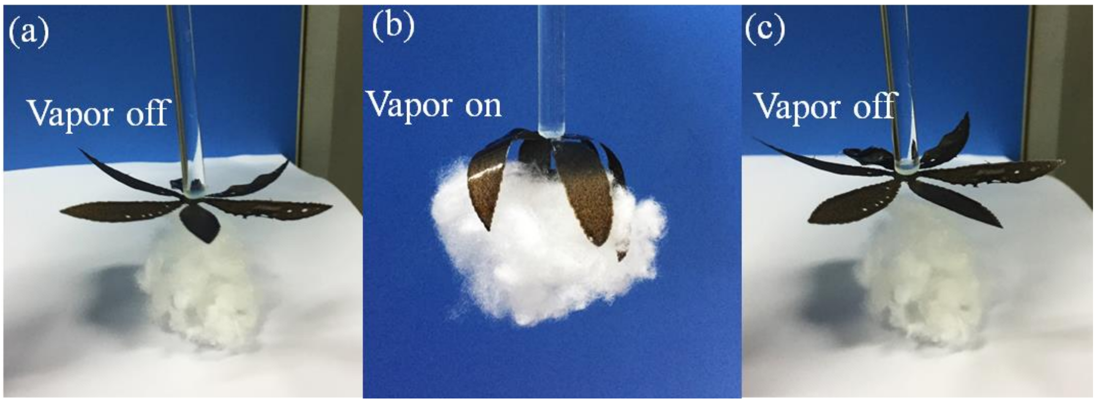

Figure S17. (a-c) Demonstration of a printed flower with six 90°/0° oriented petals as a vapor-responsive gripper to hold a cotton ball when the vapor is sequentially turned off (a), turned on (b), and turned off (c).

As demonstrated in Figure 5i, the printed strip can realize reversible shape-morphing when stimulated by water vapor. Particularly, it shows fast shape-morphing ability, which is highly demanded in application of responsive soft robotics. Thus we designed and printed a flower with six 90°/0° oriented petals, which were both fixed

on a 0°/0° oriented central disc at one end. When water vapor was turned on, these flower petals were subjected to a bending motion at another end, behaving like a closing flower (Figure S17). Light-weight objects under the flower were then grasped by the bending petals. If the vapor is keeping on, then the grasped objects can be transferred to a target location. After the vapor was turned off, these petals then quickly recovered their original planar structures, unloading the objects. By repeating the vapor on/off stimuli, the printed flower can be competent as an automatic gripper for object transportation.

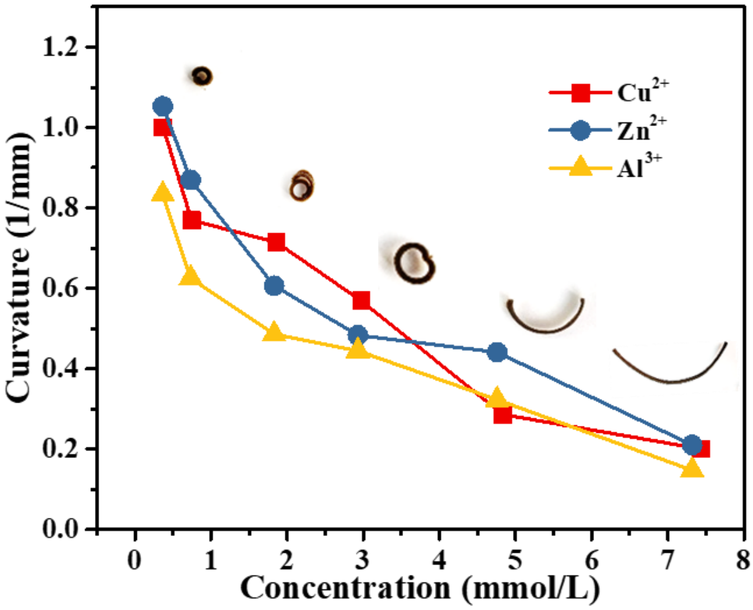

Figure S18. Bending curvatures of the 90°/0° oriented strip cross-linked with various concentrations of Cu^{2+}, Zn^{2+}, and Al^{3+} ion. Photographs showing their corresponding bending (cross-linked with Cu^{2+}) in the inset.

The shape-morphing phenomena of the printed composite in water may inspire novel applications. For demonstration, the shape-morphing behavior in water may be employed as a visualized way for detecting the concentration of heavy metal ions of

polluted water for sewage discharge. The toxic heavy metal ions in water, such as Hg^{2+}, pb^{2+}, Cu^{2+}, Cr^{2+}, etc. may impose great danger on the biological environment. Thus it is significant to monitor the concentration in a simple and efficient way instead of using an expensive and precise instrument. As suggested above, metal-ligand-interaction of the printed composite enables a tunable morphing extent in water. Thus the resultant curvature of final 3D structures offers a direct and visualized way for qualitatively detecting the concentration of metal ions. As shown in Figure S18, Cu^{2+}, Zn^{2+}, and Al^{3+} were chosen as typical toxic metal ions to cross-linking with 90°/0° oriented strip in a constant time of 10 s. As the concentration increase, the densely cross-linked network thus limits the swelling of printed composite, therefore decreasing the bending curvatures (or increasing the bending radium as shown in the inset of Figure S18). Note that the current demonstration only shows preliminary and qualitative results and the selectivity of different metal ions needs to be considered in further work.
